# Impact of Upper Limb Function on Activities of Daily Living and Quality of Life in Huntington’s Disease

**DOI:** 10.3390/jcm14010168

**Published:** 2024-12-31

**Authors:** Lucía Simón-Vicente, Jéssica Rivadeneyra, Natividad Mariscal, Laura Aguado, Irene Miguel-Pérez, Miriam Saiz-Rodríguez, Álvaro García-Bustillo, Ignacio Muñoz-Siscart, Dolores Díaz-Piñeiro, Esther Cubo

**Affiliations:** 1 Faculty of Health Sciences, Department of Physical Therapy, Occupational Therapy, Rehabilitation and Physical Medicine, Rey Juan Carlos University, 28922 Madrid, Spain; 2 Research Unit, Burgos University Hospital, 09006 Burgos, Spain; jessicajrp@hubu.es (J.R.); miriamsr@ubu.es (M.S.-R.); 3 Neurology Department, Burgos University Hospital, 09006 Burgos, Spain; namariscal@saludcastillayleon.es (N.M.); laguadog@saludcastillayleon.es (L.A.); iperezmi@saludcastillayleon.es (I.M.-P.); mcubo@saludcastillayleon.es (E.C.); 4 Health Science Department, University of Burgos, 09006 Burgos, Spain; alvarogarbu@gmail.com; 5 Psychiatry Department, Burgos University Hospital, 09006 Burgos, Spain; imunozs@saludcastillayleon.es (I.M.-S.); bmddiazp@saludcastillayleon.es (D.D.-P.)

**Keywords:** activities of daily living, manipulative dexterity, hand, Huntington’s disease

## Abstract

**Background/Objectives**: Huntington’s disease (HD) is a neurodegenerative movement disorder associated with significant disability and impairment of Activities of Daily Living (ADLs). The impact of upper limb disability on quality of life (QoL) and its influence on ADLs is not well known yet. The aim of this study was to describe the manipulative dexterity, strength, and manual eye coordination of patients with manifest and premanifest-HD compared to healthy individuals and to analyze its influence on ADLs and QoL. **Methods:** We performed an observational, cross-sectional study including 71 ambulatory participants (27 manifest-HD patients, 15 premanifest-HD, and 29 controls). We gathered sociodemographic data, as well as clinical data, including cognition (MMSE), HD motor severity (Unified HD rating scale, UHDRS-TMS), QoL (Neuro-QoL), and ADLs (HD-ADL). Hand dexterity and strength in the dominant and non-dominant hand were assessed with the Nine Hole Peg Test, Ten Neurotest, Nut and Bolt Test, dynamometry, and Late-Life FDI. Analysis of covariance (ANCOVA) models were performed to investigate differences in hand function between manifest-HD, premanifest-HD, and controls. **Results:** Manifest-HD patients had significantly worse performance in manual and finger dexterity, fine-motor coordination, and poorer handgrip strength than premanifest-HD and controls. Premanifest-HD required more time to complete the test than controls. Significant correlations were found between hand variables and Late-Life FDI, Neuro-QoL, HD-ADL, and UHDRS-TMS. **Conclusions:** HD affects manipulative dexterity and hand function in premanifest and manifest patients. Therefore, to prevent disability and decreased QoL, evaluating the progression of upper limb dysfunction in HD is important to offer the best possible therapeutic interventions.

## 1. Introduction

Huntington’s disease (HD) is a progressive, neurodegenerative disorder caused by an expansion of the CAG triplet repeat of the Huntingtin gene (≥36 repeats), located on the short arm of chromosome 4. HD is characterized by motor dysfunction, cognitive impairment, and psychiatric disturbances such as anxiety or depression [1,2].

Motor impairment in HD includes chorea, found in over 90% of cases, bradykinesia, dystonia, and rigidity, causing upper limb impairments that make the performance of manipulative, grasping, reaching, and fine motor activities difficult. Deficits in finger tapping have been reported from the early stages of the disease, and deficits in precision grip, greater grip force variability, and higher static grip force have been observed in intermediate and advanced stages [3,4]. The upper extremity’s primary role consists of the strategic positioning of hands for pushing, carrying, manipulating, lifting, pulling, and handling daily objects [5], and as the disease progresses, it has been observed that these movements become restricted, leading to a loss of independence in Activities of Daily Living (ADLs) and problems with activity and participation [6,7].

Simón-Vicente et al. [8] analyzed which occupations HD patients perceived as difficult to perform in everyday life and which occupational area the activities were related to (self-care, productivity, and leisure). They concluded that patients with HD had difficulties performing activities such as dressing, particularly tying shoelaces and shirt buttons; personal hygiene and grooming, specifically brushing teeth and shaving. Furthermore, these patients also required assistance in the ADL of feeding due to excessive food spillage and inability to maintain proximal stability [9].

In addition, motor impairment with muscle weakness and incoordination has been observed to hinder simple tasks such as cutting food or holding utensils [10]. Decreased manual dexterity, poor coordination, and reduced grip also lead to difficulties in manual tasks such as writing, holding, and operating a telephone [11]. Patients with HD mentioned the importance of preservation of identity and autonomy, emphasizing the impact of physical symptoms on their well-being and quality of life (QoL). Additionally, they reported experiences of fear, stress, and frustration as a result of a strong focus on physical symptoms, such as motor impairments and swallowing difficulties [12]. The onset of the disease leads to a decline in QoL, significantly affecting physical, emotional, and social domains [13].

Therefore, clinicians and researchers must pay attention to the progression of upper limb dysfunction in HD in order to offer the best possible therapeutic approach and consider management strategies. Although many studies have characterized motor deficits in HD [12,13,14,15,16], there is little evidence of studies that describe and predict hand function in HD patients. In order to establish a model for the interpretation of the disease and, more importantly, the best possible intervention, it is necessary to start from a solid knowledge base, including not only manual dexterity but also how it affects hand function or even participation in ADLs.

In consequence, the present study aims to describe the manipulative dexterity, strength, and manual eye coordination of patients with manifest and premanifest-HD compared to healthy individuals and to analyze its influence on ADLs and QoL.

## 2. Materials and Methods

### 2.1. Study Design

We conducted an observational, cross-sectional study including premanifest and manifest patients with HD and a healthy age- and gender-matched control group from the Neurology Department of the Burgos University Hospital in Spain. This study was approved by the Research Ethics Committee of the University Hospital of Burgos and Soria. All the procedures were conducted in accordance with Good Clinical Practice standards [17] and following the ethical principles established in the Declaration of Helsinki [18]. Participants gave their written consent and were assigned a study code to anonymize their data. Information and data were stored in a secure folder in a secure location dedicated to this study. (Certificate number: CEIM-2847).

### 2.2. Sample

A priori power analysis using G*Power version 3.1.9.7 indicated that a minimum of 14 participants per group would be required for an independent samples *t*-test to achieve 80% power in detecting a large effect, with a significance level of α = 0.05.

All participants provided written and verbal consent prior to participation. They were evaluated by neurologists with longstanding experience in HD. Premanifest patients were defined based on the Diagnostic Confidence Level (DCL) of ≤ 1 and manifest-HD >1. The DCL for HD was classified as normal (DCL = 0), nonspecific motor impairment (DCL = 1), motor impairment that may be a sign of HD (DCL = 2), motor impairment that is likely a sign of HD (DCL = 3), and motor impairment that is an unequivocal sign of HD (DCL = 4) [19].

For inclusion, participants with HD had to be 18 years or older and have received a clinical diagnosis of HD (>36 CAG repeats in the HTT gene). We excluded patients with significant cognitive impairment, inability to follow test instructions, or any concurrent musculoskeletal and/or neurological condition that could affect test performance.

### 2.3. Study Procedure

The study was conducted during one single visit at the Burgos University Hospital. The consent information from all participants was obtained before data collection. A brief interview was conducted first to gather sociodemographic information, including education (number of years), and nutrition status [body mass index (BMI)]. Next, a series of tests were conducted to measure participants’ hand function. Data collection lasted about 15–20 min for each participant.

### 2.4. Assessment Tools

Data collection included sociodemographic information such as age, sex, and handedness. HD motor severity was assessed using the Unified Huntington’s Disease Rating Scale Motor Scale [20] and Total Functional Capacity (TFC) [21]. Cognition was measured using the Mini-Mental State Exam [22]. All HD participants were evaluated using a standardized HD assessment tool, the UHDRS-TMS, which assesses motor function with 31 items using a 5-point ordinal scale ranging from 0 to 4, with 4 being the highest score, indicating the inability to perform the motor task. Total scores range from 0 to 124, with higher scores indicating greater motor impairment. Parkinsonism was calculated using the mean of the items finger taps, pronate/supinate hands, and rigidity-arm scores; dystonia using the mean of the right and left upper limb extremity dystonia scores; and finally, chorea using the mean of the right and left upper limb extremity chorea scores of the UHDRS-motor scale [20].

Hand grip strength was evaluated with the Jamar dynamometer^®^ (JAMAR PLUS+, Chicago, IL, USA). For the assessment, participants were seated, with the shoulder joint adducted and in a neutral position, the forearm in a neutral position, the elbow flexed to 90º, and the wrist slightly extended [23]. The participant was asked to squeeze the dynamometer with maximal effort for three trials, and the mean score was recorded for each upper extremity.

Manual dexterity performance was evaluated with the Nine Hole Peg Test (NHPT), a standardized assessment of hand and upper extremity function, which may offer greater sensitivity and objectivity compared to the UHDRS and has been evaluated and recommended for use. Participants had to take nine small pegs from a container, one by one, and place them into holes on a board as quickly as possible. They then removed the pegs from the holes, one by one, and replaced them back into the container. The therapist timed how long it took, first with one hand and then with the other, and the mean values of the two trials were taken for each hand [24,25].

The Ten Euro Neurotest (TEN) was used to evaluate finger dexterity. Ten coins of one euro were aligned on a straight line in the middle of a plain A4-sized paper. The paper was positioned in front of the subject, and he was asked to turn the coins as fast as possible, starting with the most distant coin. The evaluator timed how long it took to turn all the coins. This test offers a simple, quantitative measure of manual dexterity and has proven to be a reliable indicator sensitive to hand function impairments [26,27,28].

The Nut and Bolt Test (NBT) was used to measure fine motor coordination. The participants were asked to screw a nut onto a bolt in one direction, and the hand holding the bolt had to remain static. The task consisted of two different-sized (small and large) nuts and bolts and was timed for both hands with a standard stopwatch. The timer was started when the participant began turning the nut onto the bolt and ended when the nut reached the top of the bolt and could go no further. This test has recently been reported to be sensitive to changes in manual dexterity in patients with manifest and premanifest-HD [29,30].

The Late-Life Function and Disability Instrument (Late-Life FDI) was used to evaluate self-reported upper extremity function. We selected the domain of upper extremity functioning, which included items that reflected activities of the hands and arms. The participant had to assess the level of difficulty they had in carrying out the activities with five response choices: “none”, “a little”, “some”, “quite a lot”, or “cannot do”. The total raw scores were transformed to a scale from 0 to 100, with a higher score indicating better hand function [31].

The Huntington’s Disease Activities of Daily Living (HD-ADL) is a scale consisting of 17 questions related to the completion of household tasks, employment, personal care, general level of activity and interest, written and telephone communication, travel, money management, sociability, and quality of the patient’s relationships. Each item is rated on a scale from 0 (no difficulty) to 3 (maximal difficulty). The total score of the HD-ADL scale ranges from 0 (normal) to 51 (maximal limitation) [32,33].

QoL was assessed using the Short-Form Health Survey 12 (SF-12), a shortened form of the SF-36, which measures QoL and assesses the degree of well-being and functional capacity using 12 items. It evaluates two dimensions, mental and physical health, and can be administered by the researcher or self-administered. This assessment has very good test–retest reliability (ICC = 0.78) [34]. We also used the Neuro Quality of Life (Neuro-QoL), a self-report of health-related quality of life divided into 17 different domains [35]. Participants were evaluated with the “Upper Extremity Function—Fine Motor, ADL” domain, which measured the ability to carry out activities involving manual, digital, and reach-related functions. Subjects had five responses options for each question to score the degree of difficulty (1 = unable to do, 2 = with much difficulty, 3 = with some difficulty, 4 = with a little difficulty, 5 = without any difficulty) [35,36]. It is a reliable and valid assessment of physical functioning [37].

### 2.5. Statistical Analysis

Descriptive statistics were calculated for the variables. The α level for significance was *p* < 0.05, and all tests were two-tailed. We used the Shapiro–Wilk test to analyze data normality. We conducted parametric or non-parametric tests based on the normal distribution of the variables. The Clopper–Pearson method was used to calculate the confidence interval of proportions.

Group differences were analyzed using the parametric Student’s *t*-test to compare hand function among manifest-HD and controls, premanifest-HD and controls, and manifest-HD and premanifest-HD.

Analysis of covariance (ANCOVA) models were performed to investigate differences in hand function between manifest-HD, premanifest-HD, and controls after adjusting for age, sex, BMI, and cognitive state (MMSE scores) to exclude the effects of confounding factors.

Exploratory correlational analysis, using Spearman’s rho, was performed between grip strength, NHPT, TEN, and NBT for the dominant and non-dominant hand with HD-ADL, Neuro-QoL, SF-12, UHDRS-TMS, and Late-Life FDI. To categorize the level of Spearman’s correlation coefficient (*r*), we adopted the following scores: *r* < 0.40 corresponded to low correlation, *r* = 0.40–0.75 corresponded to moderate correlation, and *r* > 0.75 corresponded to high correlation [38]. Data were analyzed using SSPS v.27 statistical software.

## 3. Results

A total of 71 participants, including 44 HD gene carriers and 27 controls, were included in the study, with ages ranging from 28 to 78 years. There were 15 presymptomatic gene carriers (12 females, 80%), 29 manifest-HD (14 females, 48.28%), and 27 healthy control subjects (17 females, 63%). Demographics, test results, and clinical characteristics of the study participants are given in Table 1.

Descriptive data from the administered tests are presented in Table 2. There were significant differences between groups in grip strength, the NHPT, TEN, and NBT. People with manifest-HD had less strength in both the dominant and non-dominant hands compared with the premanifest-HD and control groups. No differences were found between premanifest-HD and controls in hand grip strength.

Regarding manual dexterity, the execution time needed to complete the task showed differences in the execution time for finger dexterity and fine-motor coordination tests in manifest-HD compared to premanifest-HD and the control group (*p* < 0.001) in all assessments. Likewise, premanifest-HD, compared to controls, required more time in the NHPT (non-dominant hand) (*p* = 0.018), in the TEN in the dominant (*p* = 0.003) and non-dominant hands (*p* = 0.025), and in the NBT in the dominant (*p* = 0.020) and non-dominant (*p* = 0.013) hands using the large bolt, and in the dominant (*p* = 0.04) and non-dominant (*p* = 0.038) hands using the small bolt. Although no statistically significant differences were found, increased grip strength in the premanifest-HD group compared to the control group was observed; however, premanifest-HD patients had the worst manipulative dexterity.

**Figure 1:**
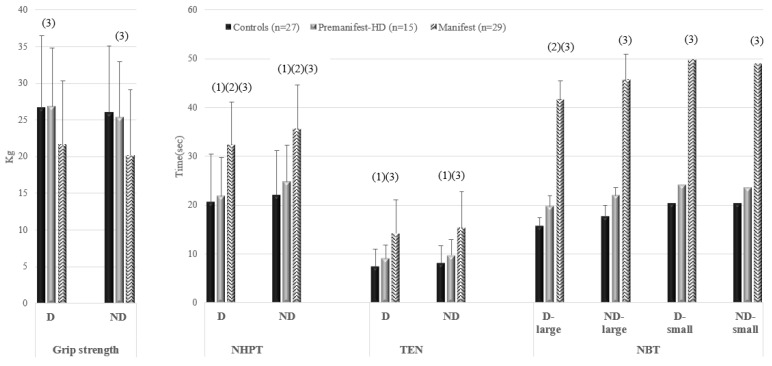
Differences between groups in the performance of different tests for the dominant and non-dominant hands after adjusting for sex, age, cognitive state, and body mass index. NHPT, Nine Hole Peg Test; TEN, Ten Neurotest; NBT, Nut and Bolt Test; D, Dominant; ND, Non-dominant. (1) Statistically significant differences between controls and premanifest-HD. (2) Statistically significant differences between manifest-HD and premanifest-HD. (3) Statistically significant differences between manifest-HD and controls.

When we compared hand function among the groups after adjusting for sex, age, cognitive state, and body mass index, we found statistically significant differences in the NHPT and NBT with the large bolt between premanifest-HD and manifest-HD, and in the NHPT, TEN, and NBT between manifest-HD and controls. Related to premanifest-HD and controls, significant differences were found in the NHPT and TEN for both dominances. In relation to grip strength, significant differences were found between manifest-HD and controls. Manifest-HD patients spent the longest time performing all tests and had the lowest grip strength (Table 3, Figure 1).

Table 4 shows the correlation among different variables with Late- Life FDI, Neuro-QoL, HD-ADL, SF-12, total score of UHDRS-TMS, and for the specific items of parkinsonism, dystonia and chorea of UHDRS-TMS.

Self-reported upper extremity function (Late-Life FDI) significantly correlated with manual dexterity performance (NHPT) in the dominant (*p* = 0.040) and in non-dominant hands (*p* = 0.004), and with finger dexterity (TEN) in the dominant hand (*p* = 0.047), indicating that worse self-reports of upper extremity function were associated with worse manual dexterity performance and finger dexterity (increased time to perform the test).

A moderate negative correlation was found between self-reported health-related QoL for the upper extremity (Neuro-QoL) and the NHPT (non-dominant hand) (*p* = 0.007), and the TEN in the dominant hand (*p* = 0.007) and non-dominant hand (*p* = 0.020) indicating that manifest-HD patients who had worse manual and finger dexterity also had worse self-reports of their health-related QoL. Non-significant correlations were found in the SF-12.

A significant positive correlation was found between HD-ADL and the NHPT in the dominant hand (*p* = 0.032) and with the TEN in the non-dominant hand (*p* = 0.047), indicating that worse finger dexterity was associated with more limitations in ADLs.

Regarding UHDRS-TMS, significant positive correlations were found between parkinsonism (right) and the NHPT in the non-dominant hand (*p* = 0.049), with the TEN in the dominant hand (*p* = 0.012) and non-dominant hand (*p* < 0.001), and with the NBT with the large nut in the dominant hand (*p* = 0.034) and non-dominant hand (*p* = 0.043) and with the small nut in the non-dominant hand (*p* = 0.010). In relation to parkinsonism (left), significant positive correlations were found with the NHPT in the dominant hand (*p* = 0.036), with the TEN in the dominant (*p* = 0.045) and non-dominant hands (*p* = 0.026), and with the NBT with the small nut in the non-dominant hand (*p* = 0.026).

Maximal dystonia in the right upper extremity correlated with the TEN in the dominant hand (*p* = 0.016), and in the left upper extremity with grip strength (dominant hand) (*p* = 0.009), and with the NBT in the dominant (*p* = 0.012) and non-dominant hands (*p* = 0.002) using the large bolt, and in the dominant (*p* = 0.018) and non-dominant (*p* = 0.001) hands using the small bolt. Chorea in the left upper extremity correlated with the TEN in the dominant (*p* = 0.021) and non-dominant hands (*p* = 0.04), and with the NBT with the large nut in the non-dominant hand (*p* = 0.041).

The total score in the UHDRS-TMS was significantly correlated with grip strength in the non-dominant hand (*p* = 0.022), with the NHPT in the dominant (*p* = 0.012) and non-dominant hands (*p* = 0.003), with the TEN in the dominant (*p* = 0.004) and non-dominant hands (*p* < 0.001), and with the NBT in the dominant (*p* = 0.025) and non-dominant hands (*p* < 0.001) using the large bolt, and in the dominant (*p* = 0.022) and non-dominant hands (*p* = 0.001) using the small bolt.

## 4. Discussion

This study aimed to describe manipulative dexterity, strength, and manual eye coordination in patients with premanifest and manifest-HD compared with healthy individuals, and to analyze its influence on ADLs and QoL. According to our findings, patients with manifest and premanifest-HD exhibit significant deficits in dexterity and manipulative function compared to controls, with longer execution times. Group differences were observed after adjusting for significant confounding factors such as nutritional status, sex, and age. Our results are consistent with the findings of other studies that showed the utility of NHPT as an objective assessment of visuomotor coordination and speed, reporting significant differences in the symptomatic HD group compared to the borderline and asymptomatic groups [24]. Similarly, different authors have shown worse performance using the Peg Insertion Score, the NBT, the TEN, and the Moneybox test in the manifest vs. controls, and tapping deficits in the manifest and premanifest stages [18,26,28,30,39,40].

Importantly, however, we found that even in the early stages of the disease, patients categorized as premanifest exhibit significant differences in their manipulative dexterity when compared to controls, and not just to symptomatic individuals, as expected. Identifying these differences and changes in manipulative dexterity from the premanifest stage is considered highly clinically relevant, as it can help detect the disease before more evident symptoms appear. This allows for early intervention, which is crucial for slowing the progression of the disease. In addition, it was shown that no differences in hand grip strength were observed between premanifest patients and controls. These results allow therapists to orient and focus their interventions on those aspects that are relevant and can have an early impact on the functionality and independence of these patients.

Our study found that QoL correlated with finger dexterity, especially with the TEN tests. QoL is a crucial parameter for accurately assessing the effectiveness of rehabilitation treatments and their overall impact on the patient. The correlation with the TEN test suggests that this tool is therefore able to highlight the impact of upper limb disability in these subjects through a test that is easy to administer, non-invasive, inexpensive, and quick. Regularly assessing manual dexterity helps professionals monitor the progression of the disease and the effectiveness of therapeutic interventions, allowing them to adjust therapies as necessary to maximize benefits. Furthermore, an accurate assessment of the functionality of the hand could have important implications in assessing the possible improvement and designing a personalized rehabilitation treatment.

One of the most frequent problems of people with HD regarding ADLs includes activities that involve manual tasks such as fastening buttons, eating, and writing, due to reduced manual dexterity, poor coordination, and reduced grip [11]. This deterioration in QoL associated with the worsening of hand dexterity seems to be progressive [36]. Our results concluded that correlations were particularly stronger with tests measuring finger dexterity, i.e., activities requiring finer motor skills. These results show that a lack of fine motor skills can limit participation in activities not only in self-care but also in communication (writing, dialing a phone number), work, and housekeeping (cooking, cleaning). Additionally, it can affect emotional and psychological well-being. Occupational performance, an important factor associated with well-being, is also highly associated with upper extremities functioning and its impact on patients’ QoL [8,27,40].

Of interest, we found that motor impairment, especially bradykinesia and dystonia, more than chorea, had an impact on hand function in people with HD in line with previous studies [27,40]. Although we do not have a compelling explanation for this finding, a possible explanation could be that chorea can be partially suppressed by patients when they are focused on a particular task. Another possibility is that chorea is more sensitive to antidopaminergic treatment, increasing hand dexterity, or, on the contrary, antidopaminergic treatment can aggravate bradykinesia and dystonia with decreased hand dexterity and consequently decreased QoL.

These findings contribute further to our characterization of upper limb function in HD and may provide guidance for future interventions to improve the functional capacity of people suffering from HD. Based on these findings, it is evident that the observed changes in upper limb functionality significantly impact the autonomy and independence of patients. These results highlight the urgent need for developing new treatment strategies aimed at improving fine motor skills. Additionally, further research is essential to explore innovative therapeutic approaches and interventions that can mitigate the adverse effects on daily activities, communication, and overall QoL. By addressing these areas, we can enhance patient outcomes and support their ability to maintain independence.

Our results should be considered in light of some limitations. The small sample size may have prevented us from detecting smaller but clinically significant effects, and it reduces the robustness of our conclusions. In addition, we have excluded patients with more advanced disease and cognitive impairment. Regarding the latter, the impact of cognitive impairment on motor dexterity, as well as the effect of antidopaminergic drugs, frequently used for controlling chorea, should be studied in more detail in future studies with larger sample sizes. Moreover, one significant limitation of this study is the inclusion of a large number of variables in the analysis with an alpha level of 0.05. Therefore, caution should be exercised when generalizing these results, and future research should consider larger sample sizes or more stringent alpha levels to mitigate this issue and to validate and extend our findings.

However, on the other hand, this is the first study, to our knowledge, that has described the manipulative dexterity, strength, and manual eye coordination of patients with manifest and premanifest-HD compared to healthy individuals and analyzed its influence on ADLs and QoL. We have included extensive multivariate analyses adjusted for confounding factors, showing promising, new results providing detailed information about disability and QoL in this vulnerable population. Since differences in hand function are already apparent from the early stages of the disease, expanding the clinical timeframe to earlier phases holds potential for investigating new biomarkers and therapeutic targets, including multidisciplinary interventions.

## 5. Conclusions

In conclusion, the findings of this study add clinically relevant information to the limited research investigating hand function, disability, and QoL among adults with manifest and premanifest-HD. Our results indicate that manipulative dexterity deficits impacting ADLs can be observed from the early stages of the disease, even in premanifest-HD patients, supporting the need for targeted interventions. Further longitudinal studies, including international studies, are needed to confirm these results.

## Figures and Tables

**Table 1:** Sociodemographic and clinical characteristics of the sample.

Total (*n* = 71)	Control Group (*n* = 27)		Premanifest-HD (*n* = 15)		Manifest-HD (*n* = 29)	
		95% CI		95% CI		95% CI
Age (years), mean ± SD	55.92 ± 13.07	[50.64, 61.2]	42 ± 9.10	[36.96, 47.04]	58.79 ± 10.17	[54.93, 62.66]
Sex						
Male, *n* (%)	11 (40.74)	[22.39, 61.2]	3 (20)	[4.33, 48.09]	15 (51.72)	[32.53, 70.55]
Female, *n* (%)	16 (59.25)	[38.80, 77.61]	12 (80)	[51.91, 95.67]	14 (48.28)	[29.45, 67.47]
Dominant upper limb						
Right, *n* (%)	23 (85.18)	[66.27, 95.81]	15 (100)	[78.20,100]	29 (100)	[88.06, 100]
Left, *n* (%)	4 (14.81)	[4.19, 33.73]	0	[0, 21.80]	0	[0, 11.94]
CAG repeat length, mean ± SD	N/A	N/A	43.38 ± 2.33	[41.98, 44.79]	42.7 ± 2.61	[41.67, 43.74]
BMI, mean ± SD	26.18 ± 4.48	[24.24, 28.11]	25.83 ± 4.33	[23.33, 28.33]	26.06 ± 4.81	[24.07, 28.05]
HD duration, mean ± SD	N/A	N/A	N/A	N/A	7.29 ± 4.44	[5.26, 9.31]
UHDRS-TMS, mean ± SD	N/A	N/A	1.93 ± 1.98	[0.79, 3.07]	31.11 ± 17.61	[24.14, 38.08]
TFC, mean ± SD	N/A	N/A	13 ± 0	[13]	8.78 ± 2.94	[7.61, 9.94]
SF-12						
Physical component, mean ± SD	50.83 ± 6.96	[43.53, 58.13]	51.73 ± 6.12	[48.2, 55.26]	45.91 ± 6.58	[43.31, 48.51]
Mental component, mean ± SD	55.30 ± 5.37	[49.66, 60.94]	52.64 ± 7.62	[48.24, 57.04]	46.89 ± 14.57	[41.13, 52.66]
MMSE, mean ± SD	29.81 ± 0.801	[29.48, 30.13]	29.87 ± 0.35	[29.67, 30.06]	27.85 ± 2.23	[26.97, 28.73]
HD-ADL, mean ± SD	N/A	N/A	1.87 ±3.4	[−0.02, 3.75]	16.59 ± 9.30	[13.05, 20.12]
Neuro-QoL, mean ± SD	N/A	N/A	39.87 ± 0.52	[39.58, 40.15]	33.45 ± 6.75	[30.88, 36.02]
Late-Life FDI (score), mean ± SD	N/A	N/A	34.6 ± 1.3	[33.88, 35.32]	25.93 ± 7.82	[22.96, 28.9]
Antidopaminergic drugs, *n* (%)	N/A	N/A	N/A	N/A	11 (37.93)	[20.67, 57.74]

UHDRS-TMS, Total Motor Score; TFC, Total Functional Capacity; SF-12, Short-Form Health Survey 12; NHPT, Nine Hole Peg Test; TEN, Ten Neurotest; NBT, Nut and Bolt Test; HD-ADL, Huntington’s Disease Activities of Daily Living; Neuro-QoL, Neuro Quality of Life; Late-Life FDI, Late-Life Function and Disability Instrument.

**Table 2:** Differences between the groups in hand function.

Variables	Controls (*n* = 27)	Premanifest-HD (*n* = 15)	Manifest-HD (*n* = 29)	P vs. C	M vs. C	M vs. P
		Mean ± SD		Cohen’s d Effect Size [95% CI]		
Grip strength (kg)						
D	26.34 ± 9.43	26.47 ± 7.80	20.4 ± 8.82	−0.015 [−0.65, 0.621]	**0.651** [0.105, 1.192]	**0.714** [0.069, 1.352]
ND	25.69 ± 8.66	24.86 ± 7.56	18.96 ± 8.90	0.1 [−0.551, 0.749]	**0.765** [0.213, 1.311]	**0.694** [0.035, 1.345]
NHPT (s						
D	20.54 ± 3.46	21.85 ± 2.76	32.96 ± 7.4	−0.405 [−1.044, 0.24]	**−2.113** [−2.77, −1.442]	**−1.779** [−2.5, −1.042]
ND	21.88 ± 3.53	24.60 ± 3.18	37.36 ± 9.2	**−0.798** [−1.453, −0.134]	**−2.184** [−2.85, −1.505]	**−1.657** [−2.366, −0.933]
TEN (s)						
D	7.35 ± 1.62	9.23 ± 2.2	14.50 ± 3.81	**−1.016** [−1.685, −0.336]	**−2.393** [−3.084, −1.689]	**−1.568** [−2.268, −0.853]
ND	8.1 ± 2.31	9.68 ± 1.59	15.62 ± 4.95	**−0.758** [−1.41, −0.096]	**−1.957** [−2.597, −1.304]	**−1.474** [−2.165, −0.769]
NBT (s)						
D-large	15.42 ± 4.9	19.8 ± 6.64	41.61 ± 14.42	**−0.784** [−1.438, −0.12]	**−2.378** [−3.068, −1.676]	**−1.761** [−2.481, −1.025]
ND-large	17.78 ± 6.09	22.25 ± 5.84	45.15 ± 20.92	**−0.845** [−1.502, −0.177]	**−1.774** [−2.395, −1.14]	**−1.315** [−1.992, −0.625]
D-small	19.83 ± 6.68	24.28 ±6.16	48.53 ± 21.29	**−0.684** [−1.33, −0.027]	**−1.778** [−2.399, −1.144]	**−1.367** [−2.078, −0.672]
ND-small	20.28 ± 5.41	24.33 ± 6.51	49.58 ± 19.45	**−0.696** [−1.345, −0.038]	**−2.005** [−2.65, −1.346]	**−1.547** [−2.245, −0.835]

NHPT, Nine Hole Peg Test; TEN, Ten Neurotest; NBT, Nut and Bolt Test; HD-ADL, Huntington’s Disease Activities of Daily Living; Neuro-QoL, Neuro Quality of Life; Late-Life FDI, Late-Life Function and Disability Instrument; P, Premanifest HD; M, Manifest HD; C, Controls; D, Dominant; ND, Non-dominant; Sec, seconds; Kg, Kilograms. Student’s *t*-test analysis. Statistically significant *p* values (*p* < 0.05) are in bold.

**Table 3:** Differences between the groups in hand function after adjusting for sex, age, cognitive state, and body mass index.

Variables	Control Group (*n* = 27)	Premanifest-HD (*n* = 15)	Manifest-HD (*n* = 29)	*p*-Value	F Value
	Mean ± SD				
Grip strength (kg)					
D	26.62 ± 9.88	26.8 ± 7.98	21.59 ± 8.76	**0.048**	3.21
ND	26 ± 9.13	25.39 ± 7.6	20.14 ± 8.99	**0.013**	4.69
NHPT (sec)					
D	20.59 ± 3.63	21.84 ± 2.86	32.30 ± 6.90	**<0.001**	18.09
ND	22.03 ± 3.63	24.73 ± 3.26	35.59 ± 7.33	**<0.001**	23.67
TEN (sec)					
D	7.4 ± 1.7	9.04 ± 2.15	14.18 ± 3.76	**<0.001**	20.79
ND	8.10 ± 2.4	9.71 ± 1.65	15.39 ± 5.24	**<0.001**	10.52
NBT (sec)					
D-large	15.73 ± 5.12	19.72 ± 6.88	41.67 ± 15.56	**<0.001**	16.89
ND-large	17.59 ± 6.29	21.99 ± 5.98	45.63 ± 22.29	**<0.001**	9.89
D-small	20.26 ± 6.89	24.12 ± 6.36	49.68 ± 22.64	**<0.001**	10.60
ND-small	20.29 ± 5.11	23.5 ± 5.86	48.89 ± 19.48	**<0.001**	13.88

NHPT, Nine Hole Peg Test; TEN, Ten Neurotest; NBT, Nut and Bolt Test; HD-ADL, Huntington’s Disease Activities of Daily Living; Neuro-QoL, Neuro Quality of Life; Late-Life FDI, Late-Life Function and Disability Instrument; P, Premanifest HD; M, Manifest HD; C, Controls; D, Dominant; ND, Non-dominant; Sec, seconds; Kg, Kilograms. Statistically significant *p* values (*p* < 0.05) are in bold. Analysis of covariance (ANCOVA), adjusting for sex, age, cognitive state, and body mass index.

**Table 4:** Intercorrelations between Late-Life FDI, Neuro-QoL, HD-ADL, SF-12, and UHDRS-TMS with hand function tests in manifest patients with HD.

	Late-Life FDI	Neuro-QoL	SF-12		HD-ADL	UHDRS-TMS						
			Physical	Mental		Parkinsonism		Dystonia		Chorea		Total
						Right	Left	RUE	LUE	RUE	LUE	
Grip strength												
D	0.22	0.069	−0.086	0.035	−0.016	−0.115	−0.228	0.066	−0.513 **	−0.063	−0.08	−0.332
ND	0.188	0.068	−0.067	0.007	−0.043	−0.26	−0.188	0.120	−0.299	−0.159	−0.153	−0.439 **
NHPT (sec)												
D	**−0.383**	−0.313	−0.211	0.125	0.4 *	0.383	0.422 **	0.206	0.299	0.092	0.152	0.476 **
ND	**−0.522**	−0.492 **	−0.09	0.077	0.362	0.398 *	0.336	0.068	0.299	−0.03	0.105	0.557 **
TEN (sec)												
D	**−0.371**	−0.526 **	−0.208	0.075	0.342	0.494 *	0.404 *	0.479**	0.351	0.334	0.458 *	0.534 **
ND	−0.246	−0.480 **	0.043	0.118	0.372 *	0.66 ***	0.445 **	0.395	0.195	0.315	0.413 *	0.619 ***
NBT (sec)												
D-large	−0.163	−0.193	0.09	0.02	0.279	0.404 *	0.343	−0.036	0.494 **	0.004	0.073	0.432 **
ND-large	−0.127	−0.24	−0.022	0.098	0.14	0.531 **	0.387	0.226	0.584 **	0.353	0.411 *	0.664 ***
D-small	0.032	−0.004	−0.055	0.104	0.201	0.354	0.34	0.014	0.468 **	0.108	0.316	0.438 **
ND-small	−0.142	−0.223	−0.122	0.194	0.292	0.506 **	0.45 **	0.344	0.61 ***	0.233	0.384	0.595 ***

UHDRS-TMS, Total Motor Score; TFC, Total Functional Capacity; SF-12, Short-Form Health Survey 12; NHPT, Nine Hole Peg Test; TEN, Ten Neurotest; NBT, Nut and Bolt Test; HD-ADL, Huntington’s Disease Activities of Daily Living; Neuro-QoL, Neuro Quality of Life; Late-Life FDI, Late-Life Function and Disability Instrument; RUE, Right Upper Extremity; LUE, Left Upper Extremity; D, Dominant; ND, Non-dominant. All manifest-HD patients were right-handed (dominant hand). Significant values in bold * *p* < 0.05; ** *p* < 0.01; *** *p* < 0.001.

## Data Availability

The data presented in this study are available upon request from the corresponding author. The data are not publicly available due to privacy or ethical restrictions.
